# Visual and Refractive Outcomes of Topography-guided Laser-assisted In Situ Keratomileusis in Virgin Eyes

**DOI:** 10.7759/cureus.2131

**Published:** 2018-01-31

**Authors:** Sharif Hashmani, Nauman Hashmani, Husna Haroon, Yusra Hashmi

**Affiliations:** 1 Department of Ophthalmology and Visual Sciences, Hashmanis Hospital; 2 Medicine, Dow Medical College, Pakistan

**Keywords:** topography guided lasik, lasik, custom lasik, topography, refractive surgery, cornea, corneal surgery

## Abstract

Introduction

We wanted to assess the efficacy, predictability, and stability of topography-guided, laser-assisted in situ keratomileusis (TGL) on normal untreated eyes with a preoperative best corrected visual acuity (BCVA) of 20/20.

Methods

This was a retrospective, non-randomized, and single arm study evaluating the outcomes of TGL in eyes with a preoperative BCVA of 20/20. We included 50 eyes of 50 patients who presented to the Hashmanis Hospital, Pakistan and were followed for six months postoperatively. All eyes underwent treatment using the Alcon Wavelight Allegro Topolyzer (Alcon Laboratories, Inc., TX, USA).

Results

The mean preoperative sphere, cylinder, and spherical equivalent (SE) values were -4.3 ± 1.6 dioptres (D), -1.0 ± 0.8 D, and -4.8 ± 1.8 D. On day one these values were 0.2 ± 0.8 D, -0.5 ± 0.3, and  0.3 ± 0.8, respectively, and on month six they were -0.1 ± 0.6, -0.5 ± 0.3, and -0.4 ± 0.6 D, respectively. On postoperative day one and month six, 86% and 94% of eyes had a UCVA of 20/20 or better, respectively. Two eyes (4%) had an SE change of greater than 0.5 D from three to six months.

Conclusion

Our study demonstrates good efficacy, predictability, and stability of eyes undergoing TGL with a follow-up of six months.

## Introduction

Laser-assisted in situ keratomileusis (LASIK) has improved significantly in the last decade and is currently the most frequently performed refractive surgery [1, 2]. It is both safe and effective for a wide variety of vision problems with a satisfaction rate of over 95% [3]. There are several types of LASIK techniques including wavefront optimized (WO), wavefront guided (WG), and topography guided (TG) [4].

Several studies have shown that TG LASIK has superior outcomes when compared to other LASIK modalities [5]. It provides superior outcomes when used in corneas with extensive scarring or high aberrations where wavefront aberrometry cannot be done [6, 7]. The technique is also effective for patients in terms of wound healing, flap induced aberrations as well as ablation predictability when compared to the theoretical models of wavefront maps [8]. Additionally, studies have shown promising outcomes in virgin eyes in terms of uncorrected visual acuity (UCVA), best corrected visual acuity (BCVA), and refractive cylinder [9, 10].

There are a limited number of studies in the world that have evaluated the outcomes of TG LASIK. Even fewer studies have evaluated the results of this technique on virgin eyes. Furthermore, no such study has been done in Pakistan. Therefore we wanted to evaluate the visual and refractive outcomes of TG LASIK on eyes that have not undergone a refractive procedure before.

## Materials and methods

Patients

This retrospective, single arm, and nonrandomized study was reviewed and approved by the ethics committee of the Hashmanis Hospital. On the basis of eligibility criteria, 50 eyes of 50 patients who had follow-up records of at least six months were included. The study period lasted for one year from June 2016 to July 2017. All surgeries were performed by the same surgeon (SH) at the two centres of the Hashmanis Hospital, Karachi, Pakistan. Furthermore, a written informed consent was obtained from each refractive surgery candidate before the LASIK procedure.

Inclusion and exclusion criteria

The study protocol [11, 12] and reasons for exclusion [13] have been discussed in previous studies. Included in the study were patients aged 18 years or over who sought LASIK eye surgery for spectacle independence. All patients had a stable refractive error with myopia of less than -9.00 D. Anyone with a central corneal thickness (CCT) of less than 480 μm or an estimated residual stromal bed thickness of less than 250 μm were excluded. Those with active or residual ocular disease, retinal pathologies, significant dry eyes with a Schirmer’s test two value of less than 2 mm, and a history of previous corneal surgery were excluded from the study. Also excluded were pregnant or lactating women, immunocompromised individuals, those who had diabetes mellitus or autoimmune disease or were currently on systemic corticosteroids or immunosuppressants. Patients were asked to sign a written informed consent before the surgery.

Prior to the surgical procedure, a complete ocular examination was performed in each patient that included measurement of UCVA, BCVA, refraction using an auto-refractometer (KR-800; Topcon Medical Systems, Inc., Fukuoka, Japan), dilated fundus exam, anterior segment slit-lamp biomicroscopy, corneal topography, ultrasonic pachymetry, and keratometry. Patients were asked to discontinue wearing soft contact lenses one week prior to the screening.

We used the topography-guided custom ablation treatment (T-CAT), which included topography, keratometry, and pupilometry; this was done using the Allegro topolyzer (Alcon Laboratories, Inc., TX, USA). We selected images covering an adequate area from two to eight topography images and these were transferred to the study computer. Then we added the refraction data and asphericity correction to the computer. The software calculated the ablation pattern using the data provided; this was subsequently transferred to the laser platform.

Procedure and postoperative care

Patients were scheduled for topography-guided simultaneous bilateral LASIK procedure. The same surgeon operated each eye under topical anaesthesia; only one eye was included in the final analysis. The LASIK platform used was Allegretto Wave Eye-Q Laser System (Alcon surgical, Inc., TX, USA) with Allegro topolyzer topography system and T-CAT treatment planning software. The flap was created using a wavelight FS200 Laser machine (Alcon, Ft Worth, TX, USA) with an intended flap thickness of 120 μm. Stromal ablation was performed using Wavelight EX 500 machine (Alcon, Ft Worth, TX, USA), which was topography guided. The Pocket II ultrasonic pachymeter (Quantel Medical, Inc., Bozeman, MT, USA) was used to measure the central corneal thickness, both pre- and intraoperatively. The central corneal thickness was measured on the apex of the cornea prior to surgery. After the creation of the flap, CCT was measured a second time in order to assess the underlying stromal thickness. The flap was raised using a tissue separator, and a balanced solution was used to irrigate the eyes once the procedure was completed and the flap was repositioned.

All patients were given standard postoperative treatment consisting of a dexamethasone/ tobramycin combination four times every day for 10 days. Additionally, they were instructed to use moxifloxacin eye drops four times daily for 10 days and artificial tears four times daily for three weeks.

The patients were evaluated on day one, week one, and month one, three, and six postoperatively to assess their refractive outcomes in terms of UCVA, sphere, cylinder, and spherical equivalent (SE). Emmetropia was the refractive target in all cases; however, this was dependent on the keratometry reading. The efficacy index was defined as the ratio of the mean postoperative UCVA to the mean preoperative BCVA.

Statistical analysis

We used the statistical package for the social sciences (SPSS; SPSS Inc., Chicago, IL, USA) to analyse the data. We used descriptive statistics to calculate the mean and standard deviations of all data. Subsequently, we used both SPSS and Microsoft Excel (Microsoft Corp., Redmond, WA, USA) to create the various graphs.

## Results

**Figure 1:**
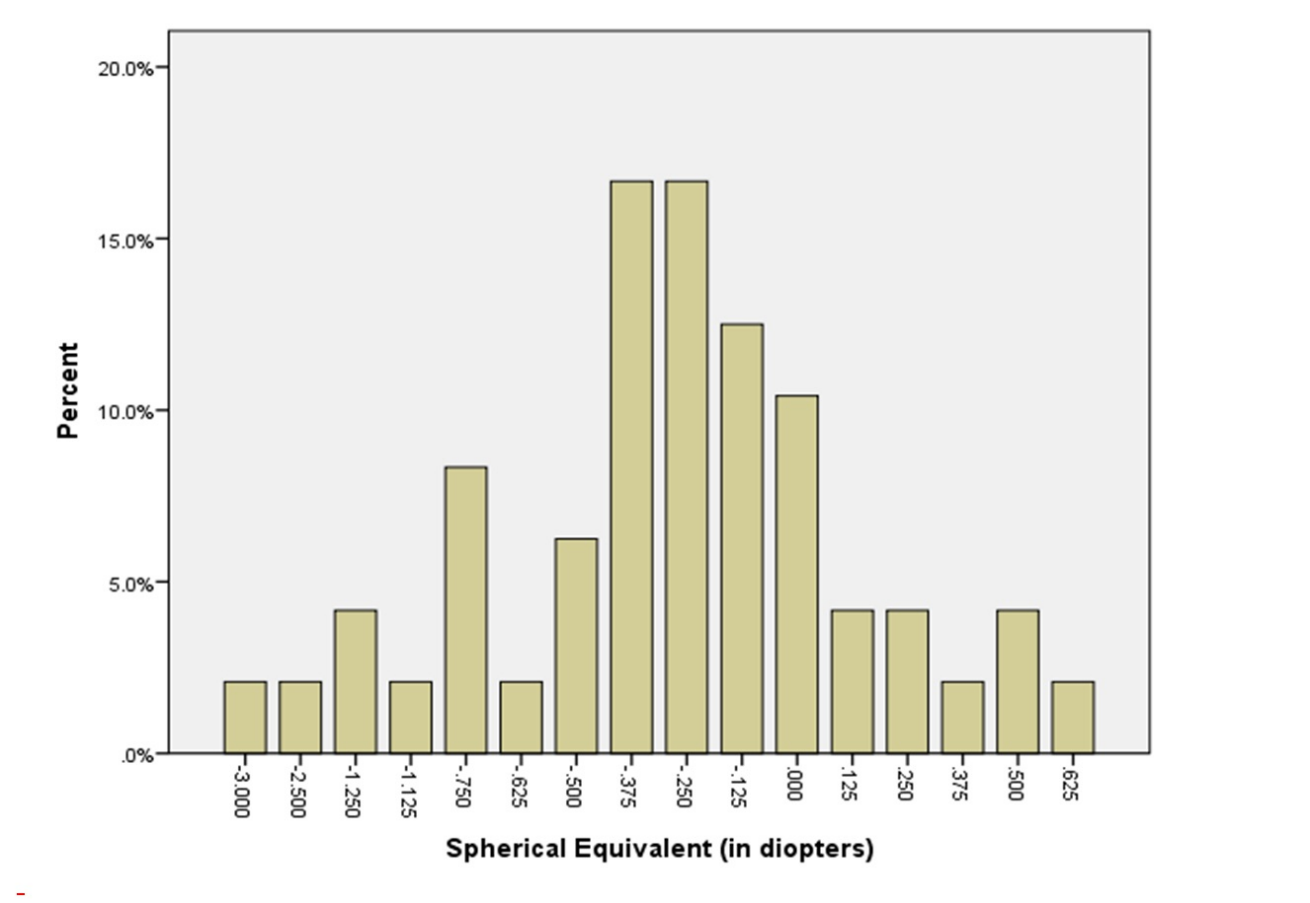
Refractive outcomes

**Figure 2:**
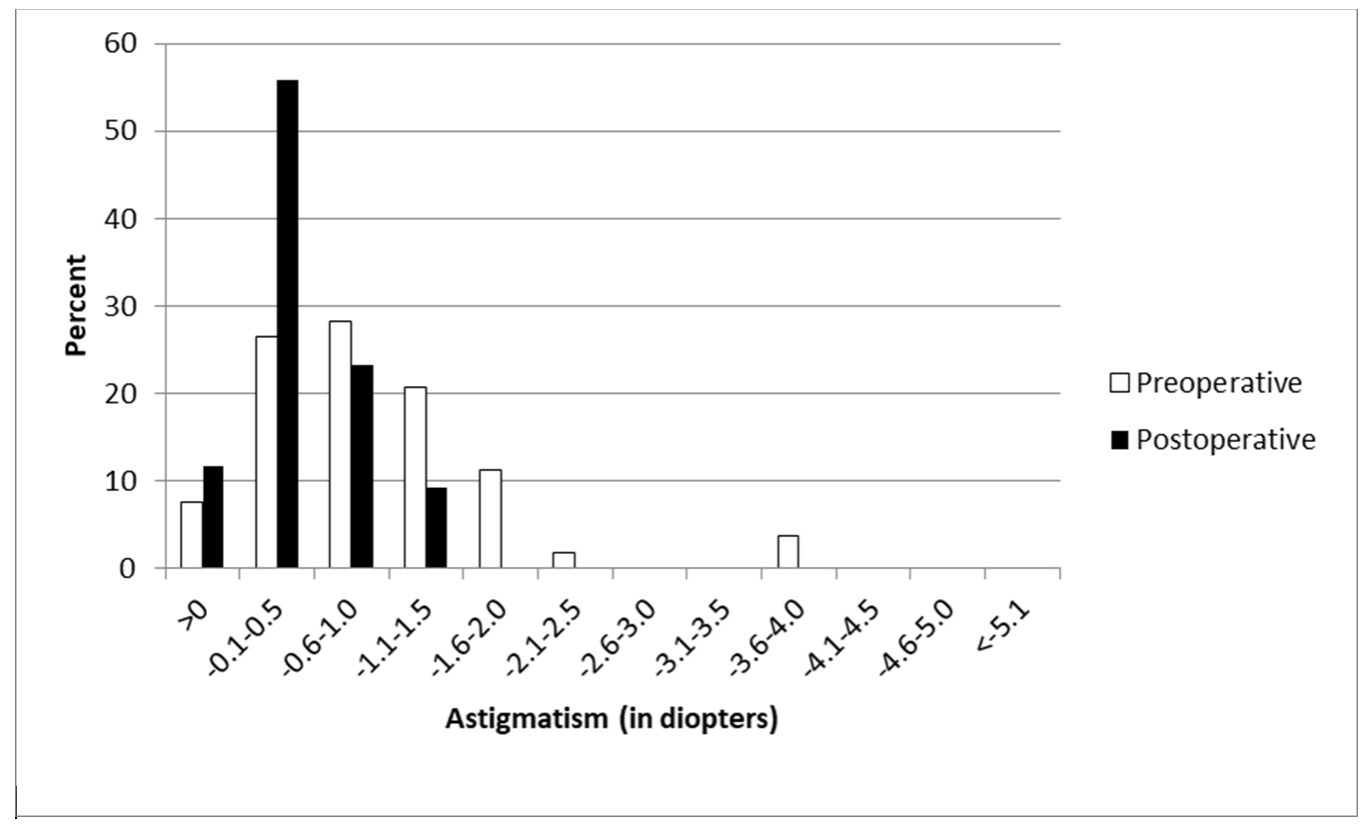
Distribution of astigmatism at month six

The mean age of eight men (16%) and 42 women (84%) included in the study was 26.4 ± 4.6 years. Table 1 shows the refractive outcomes of the eyes treated. The mean preoperative sphere, cylinder, and spherical equivalent values were -4.3±1.6 D, -1.0±0.8 D and -4.7±1.6 D, respectively. At the sixth postoperative month, these were -0.1 ± 0.6 D, -0.5 ± 0.3 D, and -0.4 ± 0.6 D, respectively. Figures 1-2 display the refractive outcomes of this modality.

**Table 1:** General characteristics Abbreviations: Y=years, M=male, F=female, D=diopters Data presented as mean ± SD

Variable	Preoperative	Post day 1	Post week 1	Post month 1	Post month 3	Post month 6
Age (Y)	26.4±4.6					
Gender (M/F)	8/42					
Sphere (D)	-4.3±1.6	0.2±0.8	0.0±0.6	-0.0±0.5	-0.0±0.6	-0.1±0.6
Cylinder (D)	-1.0±0.8	-0.5±0.3	-0.5±0.3	-0.5±0.3	-0.5±0.3	-0.5±0.3
Spherical Equivalent (D)	-4.7±1.6	0.0±0.8	-0.2±0.5	-0.2±0.5	-0.3±0.6	-0.4±0.6

Efficacy

**Figure 3:**
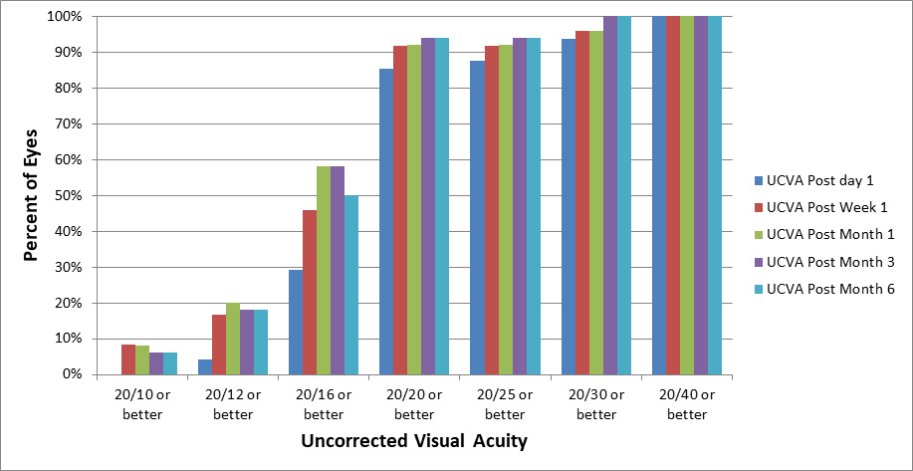
Visual outcomes throughout various postoperative checkups

At the six month follow-up, the mean efficacy index was 1.47. On the first postoperative day, 43 eyes (86%) had a cumulative UCVA of 20/20 or better. At six months, however, the cumulative UCVA was 20/20 or better in 47 eyes (94%; Figure 3).

Predictability

**Figure 4:**
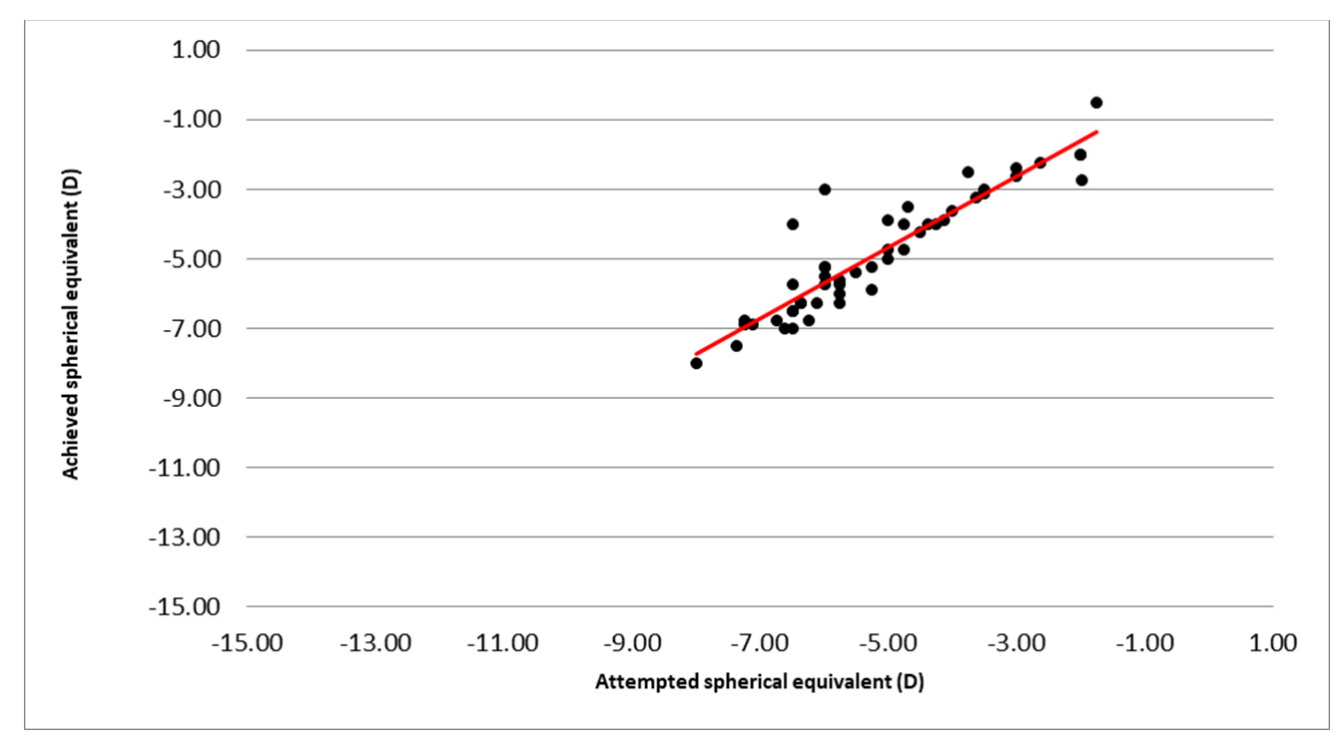
Predictability of measurements

The postsurgical refractive outcome at six months showed good predictability of this ablation profile, with 45 eyes (90%) achieving a mean spherical equivalent within 1.00 of the intended value (Figure 4).

Stability

**Figure 5:**
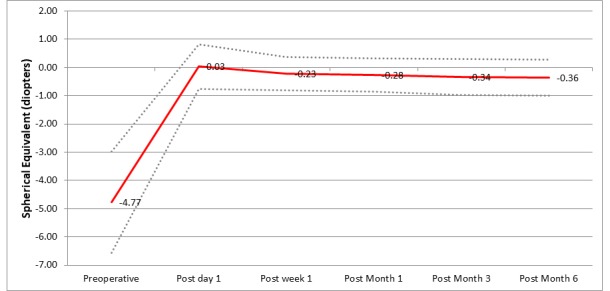
Stability of the procedure

Out of the 50 eyes treated, two eyes (4%) had a manifest refraction spherical equivalent (MRSE) change of more than 0.5 D from three to six months (Figure 5).

## Discussion

Popular LASIK modalities include WO, WG, and TG. WO LASIK is one of the most popular and accepted current modalities, which takes into account the patient’s refraction. While this modality accounts for lower order aberrations, higher order aberrations (HOAs) are never considered. WG LASIK uses wavefront data acquired from an aberrometer and while this modality takes into account both refraction and HOAs, its sole reliance on a wavefront aberrometer poses some limitations [14]. TG LASIK uses data from refraction and corneal topography to create a smooth and regular cornea and can alleviate some of the limitations of WG LASIK.

Firstly, wavefront measurements are limited by the pupil and therefore peripheral measurements are not taken into consideration. Secondly, eyes undergo pupil centroid shift causing visual quality deterioration. Topographic calculations are not dependent on the pupil size or shape and they cover the peripheral cornea as well as the central. This is an important advantage of TG LASIK as peripheral corneal irregularities are responsible for the majority of HOAs. The use of TG LASIK in eyes with small or off-centre optical zones has been shown by previous studies [15-17].

Furthermore, opacities in the cornea, lens, and vitreous can lead to flawed data. Opacities can lead to light scattering, which can influence the measurements provided by the aberrometer. Highly aberrated eyes can have a similar effect as well. TG LASIK is not influenced by these opacities and has been successfully used in eyes after trauma [10], flap interface complications [18], and ectatic [19] and keratonic eyes after corneal cross linking.

TG LASIK was once used only for the purpose of treating corneas that were irregular or pathological. According to early reports, TG LASIK has proven to be successful in regularisation of the cornea and improving vision after corneal trauma, keratoplasty, and various laser ablation problems [10, 20]. Some studies, however, show an overall better outcome as well as fewer adverse effects after TG LASIK compared to other, more commonly used, ablation patterns in virgin eyes [21-23].

Our study was a single arm study and was unable to compare results with other modalities like WO and WG LASIK. There is controversy in the literature regarding the superior visual outcomes of the modalities. El Awady et al. [23] and Jain et al. [24] found superior BCVA postoperatively in the TG LASIK arm when compared to WO LASIK. However, Shetty et al. [25] found no difference in this parameter. A comparable disparity is also seen when comparing the two custom ablation systems.

It was interesting to note the regression of the SE in our study when compared to the study by Stulting et al. [14]. Our study had a mean myopic regression of approximately 0.39 D over six months. In comparison, Stulting et al. reported one of only 0.06 D. While slight differences may be present due to the difference in the method of recording the SE, we believe there are underlying factors causing this that we were unable to record. We recommend further research into this particular topic.

Multiple studies have shown fewer HOA inductions in TG LASIK when compared to other modalities, [24, 25] especially in eyes that had a high number of aberrations [7]; the reason for this has been discussed previously. However, we recommend exploring the indications of the separate custom ablation modalities as it is unlikely that one modality will be superior to the other in all situations.

There were a few limitations in our study. Firstly, this was a single arm study and therefore no direct comparisons were made with other modalities. Secondly, we were unable to perform a subjective assessment of contrast sensitivity and HOAs due to a technical limitation at the facility. Lastly, this was a retrospective study and all associated limitations must be considered.

## Conclusions

Our study demonstrates good efficacy, predictability, and stability of eyes undergoing TG LASIK with a follow-up of six months.

## References

[1] Varley GA, Huang D, Rapuano CJ (2004). LASIK for hyperopia, hyperopic astigmatism, and mixed astigmatism: a report by the American Academy of Ophthalmology. Ophthalmology.

[2] Pillar A, Krueger R (2016). Advances in refractive surgery: June 2014 to July 2015. Asia Pac J Ophthalmol (Phila).

[3] Solomon KD, de Castro LEF, Sandoval HP (2009). LASIK world literature review: quality of life and patient satisfaction. Ophthalmology.

[4] Krueger RR, Rocha KM (2008). Introduction to wavefront-optimized, wavefront-guided, and topography-guided customized ablation: fifth year in review. J Refract Surg.

[5] Kanellopoulos AJ, Kahn J (2012). Topography-guided hyperopic LASIK with and without high irradiance collagen cross-linking: initial comparative clinical findings in a contralateral eye study of 34 consecutive patients. J Refract Surg.

[6] Lin DT, Holland S, Tan JC, Moloney G (2012). Clinical results of topography-based customized ablations in highly aberrated eyes and keratoconus/ectasia with cross-linking. J Refract Surg.

[7] Lin DT, Holland SP, Rocha KM, Krueger RR (2008). Method for optimizing topography-guided ablation of highly aberrated eyes with the ALLEGRETTO WAVE excimer laser. J Refract Surg.

[8] Tan J, Simon D, Mrochen M, Por YM (2012). Clinical results of topography-based customized ablations for myopia and myopic astigmatism. J Refract Surg.

[9] Jankov MR, Panagopoulou SI, Tsiklis NS (2006). Topography-guided treatment of irregular astigmatism with the wavelight excimer laser. J Refract Surg.

[10] Knorz MC, Jendritza B (2000). Topographically-guided laser in situ keratomileusis to treat corneal irregularities. Ophthalmology.

[11] Hashmani N, Hashmani S, Ramesh P (2017). A comparison of visual outcomes and patient satisfaction between photorefractive keratectomy and femtosecond laser-assisted in situ keratomileusis. Cureus.

[12] Hashmani S, Hashmani N, Rajani H (2017). Comparison of visual acuity, refractive outcomes, and satisfaction between LASIK performed with a microkeratome and a femto laser. Clin Ophthalmol.

[13] Hashmani S, Hashmani N, Kumar S (2017). Reasons for refusing laser-assisted in situ keratomileusis in a Pakistani population. Cureus.

[14] Stulting RD, Fant BS, Group T-CS (2016). Results of topography-guided laser in situ keratomileusis custom ablation treatment with a refractive excimer laser. J Cataract Refract Surg.

[15] Kanellopoulos A (2005). Topography-guided custom retreatments in 27 symptomatic eyes. J Refract Surg.

[16] Kymionis GD, Panagopoulou SI, Aslanides I (2004). Topographically supported customized ablation for the managment of decentered laser in situ keratomileusis. Am J Ophthalmol.

[17] Hafezi F, Mrochen M, Seiler T (2005). Two-step procedure to enlarge small optical zones after photorefractive keratectomy for high myopia. J Cataract Refract Surg.

[18] Chen X, Stojanovic A, Zhou W (2012). Transepithelial, topography-guided ablation in the treatment of visual disturbances in LASIK flap or interface complications. J Refract Surg.

[19] Kanellopoulos AJ, Binder PS (2011). Management of corneal ectasia after LASIK with combined, same-day, topography-guided partial transepithelial PRK and collagen cross-linking: the athens protocol. J Refract Surg.

[20] Wiesinger-Jendritza B, Knorz MC, Hugger P, Liermann A (1998). Laser in situ keratomileusis assisted by corneal topography. J Cataract Refract Surg.

[21] Farooqui MA, Al-Muammar AR (2006). Topography-guided CATz versus conventional LASIK for myopia with the NIDEK EC- 5000: a bilateral eye study. J Refract Surg.

[22] Dougherty PJ, Waring G, Chayet A (2008). Topographically guided laser in situ keratomileusis for myopia using a customized aspherical treatment zone. J Cataract Refract Surg.

[23] El Awady HE, Ghanem AA, Saleh SM (2011). Wavefront-optimized ablation versus topography-guided customized ablation in myopic LASIK: comparative study of higher order aberrations. Ophthalmic Surg Lasers Imaging.

[24] Jain AK, Malhotra C, Pasari A (2016). Outcomes of topography-guided versus wavefront-optimized laser in situ keratomileusis for myopia in virgin eyes. J Cataract Refract Surg.

[25] Shetty R, Shroff R, Deshpande K (2017). A prospective study to compare visual outcomes between wavefront-optimized and topography-guided ablation profiles in contralateral eyes with myopia. J Refract Surg.

